# *N*-Benzyl-*N*-methyl­morpholinium chloride

**DOI:** 10.1107/S1600536808040506

**Published:** 2008-12-06

**Authors:** Yan-Jiang Bian

**Affiliations:** a Faculty of Chemistry and Materials Science, Langfang Teachers’ College, Hebei, Langfang 065000, People’s Republic of China

## Abstract

In the title compound, C_12_H_18_NO^+^·Cl^−^, the cations and anions are inter­connected by weak C—H⋯Cl hydrogen bonds. The morpholine ring system adopts a chair conformation.

## Related literature

For general background to ionic liquids, see: Abedin *et al.* (2004 ▶, 2005 ▶); Kim *et al.* (2005 ▶, 2006 ▶).
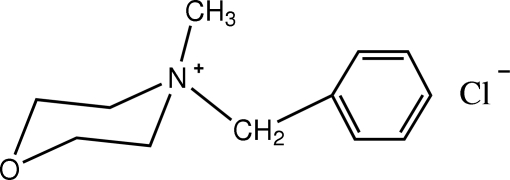


## Experimental

### 

#### Crystal data

C_12_H_18_NO^+^·Cl^−^
*M* *_r_* = 227.72
Orthorhombic, 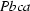

*a* = 9.8693 (8) Å
*b* = 9.5732 (8) Å
*c* = 24.989 (2) Å
*V* = 2361.0 (4) Å^3^
*Z* = 8
Mo *K*α radiation
μ = 0.30 mm^−1^
*T* = 113 (2) K
0.22 × 0.20 × 0.16 mm

#### Data collection

Rigaku Saturn CCD area-detector diffractometer
Absorption correction: multi-scan (*CrystalClear*; Rigaku/MSC, 2005 ▶) *T*_min_ = 0.937, *T*_max_ = 0.954
23984 measured reflections
2806 independent reflections
2658 reflections with *I* > 2σ(*I*)
*R*_int_ = 0.045

#### Refinement

*R*[*F* ^2^ > 2σ(*F* ^2^)] = 0.039
*wR*(*F* ^2^) = 0.103
*S* = 1.14
2806 reflections
137 parameters
H-atom parameters constrained
Δρ_max_ = 0.26 e Å^−3^
Δρ_min_ = −0.37 e Å^−3^

### 

Data collection: *CrystalClear* (Rigaku/MSC, 2005 ▶); cell refinement: *CrystalClear*; data reduction: *CrystalClear*; program(s) used to solve structure: *SHELXS97* (Sheldrick, 2008 ▶); program(s) used to refine structure: *SHELXS97* (Sheldrick, 2008 ▶); molecular graphics: *SHELXTL* (Sheldrick, 2008 ▶); software used to prepare material for publication: *SHELXTL*.

## Supplementary Material

Crystal structure: contains datablocks I, global. DOI: 10.1107/S1600536808040506/bt2824sup1.cif

Structure factors: contains datablocks I. DOI: 10.1107/S1600536808040506/bt2824Isup2.hkl

Additional supplementary materials:  crystallographic information; 3D view; checkCIF report

## Figures and Tables

**Table 1:** Hydrogen-bond geometry (Å, °)

*D*—H⋯*A*	*D*—H	H⋯*A*	*D*⋯*A*	*D*—H⋯*A*
C3—H3*A*⋯Cl1^i^	0.99	2.70	3.6610 (14)	163
C5—H5*A*⋯Cl1^ii^	0.99	2.74	3.6304 (14)	150
C5—H5*B*⋯Cl1	0.99	2.63	3.5373 (14)	152
C9—H9⋯Cl1^iii^	0.95	2.80	3.5599 (16)	138
C12—H12*A*⋯Cl1^ii^	0.98	2.70	3.6085 (14)	155
C12—H12*B*⋯Cl1	0.98	2.78	3.6566 (14)	149
C12—H12*C*⋯Cl1^iv^	0.98	2.68	3.6380 (14)	166

Symmetry codes: (i) 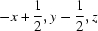; (ii) 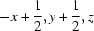; (iii) 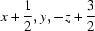; (iv) 
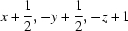
.

**Fig. 1.**
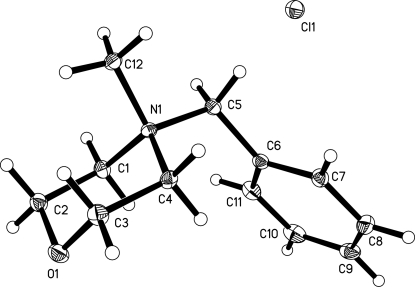
A view of the molecular structure of the title compund, showing the atom-numbering scheme. Dispacement ellipsoids are drawn at the 30% probability level.

**Fig. 2.**
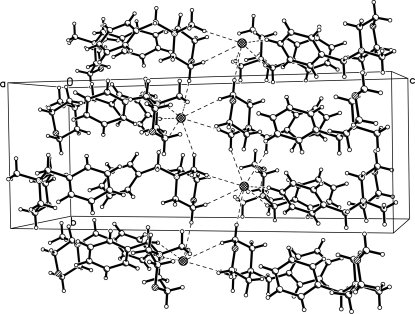
The packing of the title compound, showing hydrogen-bond interactions as dashed lines.

## supplementary crystallographic information

### Comment

Quaternary morpholine halides are valuable precursors for the preparation of ionic liquids (ILs) by ion metathesis (Kim *et al.*, 2005). The excellent conductivity, broad electrochemical window, thermal stability, and low volatility of ILs have made them promising media for electrochemical processes (Abedin *et al.*, 2004; Abedin *et al.*, 2005). In particular, ILs based on the morpholinium cation are favored because  of their low cost, easy synthesis, and electrochemical stability (Kim *et al.*, 2006). We report here a new example structure of this class.

The molecular structure of the title compound is illustrated in Fig. 1. The morpholine unit adopts a chair conformation. The bond distances and angles in the cation are normal within experimental error.

The crystal packing  is illustrated in Fig. 2. The Cl^-^anion is involved in weak C—H···Cl hydrogen bonds. Each cation forms a network of weak C—H···Cl hydrogen bonds to surrounding chloride ions.

### Experimental

Under vigorous stirring, benzyl chloride (0.12 mol) was added to a solution of 4-methylmorpholine (0.1 mol) in 20 ml of acetonitrile. The mixture was stirred at 60 °C for 5 h. The solvent was removed under reduced pressure. The remaining brownish, viscous liquid crystallized slowly at room temperature in ethanol and acetone [1/20(*v*/*v*)].

### Refinement

H atoms were included in the refinement in the riding and rotation model approximation, with C–H = 0.96–0.97 Å and *U*_iso_ (H) = 1.2 *U*_eq_(C) or *U*_iso_(H) = 1.5*U*_eq_(C_methyl_).

### Crystal data

**Table:**

C_12_H_18_NO^+^·Cl^−^	*D*_x_ = 1.281 Mg m^−^^3^
*M**_r_* = 227.72	Mo *K*α radiation, λ = 0.71070 Å
Orthorhombic, *P**b**c**a*	Cell parameters from 5341 reflections
*a* = 9.8693 (8) Å	θ = 1.6–27.9°
*b* = 9.5732 (8) Å	µ = 0.30 mm^−^^1^
*c* = 24.989 (2) Å	*T* = 113 K
*V* = 2361.0 (4) Å^3^	Prism, colorless
*Z* = 8	0.22 × 0.20 × 0.16 mm
*F*(000) = 976	

### Data collection

**Table:**

Rigaku Saturn CCD area-detector diffractometer	2806 independent reflections
Radiation source: rotating anode	2658 reflections with *I* > 2σ(*I*)
confocal	*R*_int_ = 0.045
Detector resolution: 7.31 pixels mm^-1^	θ_max_ = 27.9°, θ_min_ = 1.6°
ω and φ scans	*h* = −12→12
Absorption correction: multi-scan (*CrystalClear*; Rigaku/MSC, 2005)	*k* = −12→12
*T*_min_ = 0.937, *T*_max_ = 0.954	*l* = −32→32
23984 measured reflections	

### Refinement

**Table:**

Refinement on *F*^2^	Primary atom site location: structure-invariant direct methods
Least-squares matrix: full	Secondary atom site location: difference Fourier map
*R*[*F*^2^ > 2σ(*F*^2^)] = 0.039	Hydrogen site location: inferred from neighbouring sites
*wR*(*F*^2^) = 0.103	H-atom parameters constrained
*S* = 1.14	*w* = 1/[σ^2^(*F*_o_^2^) + (0.0504*P*)^2^ + 0.8783*P*] where *P* = (*F*_o_^2^ + 2*F*_c_^2^)/3
2806 reflections	(Δ/σ)_max_ = 0.001
137 parameters	Δρ_max_ = 0.26 e Å^−^^3^
0 restraints	Δρ_min_ = −0.37 e Å^−^^3^

### Special details

**Table:**

Geometry. All e.s.d.'s (except the e.s.d. in the dihedral angle between two l.s. planes) are estimated using the full covariance matrix. The cell e.s.d.'s are taken into account individually in the estimation of e.s.d.'s in distances, angles and torsion angles; correlations between e.s.d.'s in cell parameters are only used when they are defined by crystal symmetry. An approximate (isotropic) treatment of cell e.s.d.'s is used for estimating e.s.d.'s involving l.s. planes.
Refinement. Refinement of *F*^2^ against ALL reflections. The weighted *R*-factor *wR* and goodness of fit *S* are based on *F*^2^, conventional *R*-factors *R* are based on *F*, with *F* set to zero for negative *F*^2^. The threshold expression of *F*^2^ > σ(*F*^2^) is used only for calculating *R*-factors(gt) *etc*. and is not relevant to the choice of reflections for refinement. *R*-factors based on *F*^2^ are statistically about twice as large as those based on *F*, and *R*- factors based on ALL data will be even larger.

### Fractional atomic coordinates and isotropic or equivalent isotropic displacement parameters (Å^2^)

**Table:**

	*x*	*y*	*z*	*U*_iso_*/*U*_eq_	
Cl1	0.06425 (3)	0.24262 (3)	0.579326 (12)	0.01816 (12)	
O1	0.67612 (10)	0.15043 (10)	0.57658 (4)	0.0243 (2)	
N1	0.44588 (10)	0.33344 (11)	0.59493 (4)	0.0150 (2)	
C1	0.59109 (13)	0.37790 (14)	0.60368 (5)	0.0188 (3)	
H1A	0.6158	0.3622	0.6416	0.023*	
H1B	0.5997	0.4791	0.5962	0.023*	
C2	0.68808 (14)	0.29772 (15)	0.56803 (6)	0.0215 (3)	
H2A	0.6683	0.3193	0.5301	0.026*	
H2B	0.7822	0.3275	0.5757	0.026*	
C3	0.54189 (14)	0.10657 (14)	0.56303 (6)	0.0224 (3)	
H3A	0.5349	0.0039	0.5668	0.027*	
H3B	0.5228	0.1307	0.5252	0.027*	
C4	0.43833 (13)	0.17620 (14)	0.59888 (5)	0.0178 (3)	
H4A	0.3464	0.1448	0.5886	0.021*	
H4B	0.4543	0.1475	0.6364	0.021*	
C5	0.35303 (13)	0.40397 (14)	0.63597 (5)	0.0183 (3)	
H5A	0.3710	0.5057	0.6352	0.022*	
H5B	0.2580	0.3899	0.6245	0.022*	
C6	0.36606 (13)	0.35467 (14)	0.69294 (5)	0.0181 (3)	
C7	0.28094 (14)	0.24900 (14)	0.71151 (6)	0.0210 (3)	
H7	0.2193	0.2053	0.6876	0.025*	
C8	0.28540 (15)	0.20693 (17)	0.76477 (6)	0.0278 (3)	
H8	0.2272	0.1346	0.7770	0.033*	
C9	0.37464 (16)	0.27038 (17)	0.79996 (6)	0.0295 (3)	
H9	0.3784	0.2411	0.8363	0.035*	
C10	0.45814 (16)	0.37636 (18)	0.78209 (6)	0.0301 (4)	
H10	0.5188	0.4203	0.8063	0.036*	
C11	0.45410 (14)	0.41926 (16)	0.72890 (6)	0.0240 (3)	
H11	0.5115	0.4927	0.7171	0.029*	
C12	0.39548 (14)	0.38154 (14)	0.54104 (5)	0.0193 (3)	
H12A	0.3981	0.4838	0.5394	0.029*	
H12B	0.3021	0.3494	0.5358	0.029*	
H12C	0.4534	0.3427	0.5129	0.029*	

### Atomic displacement parameters (Å^2^)

**Table:**

	*U*^11^	*U*^22^	*U*^33^	*U*^12^	*U*^13^	*U*^23^
Cl1	0.01759 (19)	0.01818 (19)	0.01873 (18)	0.00039 (11)	−0.00043 (10)	0.00056 (11)
O1	0.0205 (5)	0.0204 (5)	0.0320 (5)	0.0046 (4)	0.0022 (4)	0.0013 (4)
N1	0.0162 (5)	0.0133 (5)	0.0156 (5)	0.0002 (4)	−0.0003 (4)	0.0007 (4)
C1	0.0169 (6)	0.0186 (6)	0.0209 (6)	−0.0027 (5)	0.0002 (5)	−0.0015 (5)
C2	0.0178 (6)	0.0211 (7)	0.0257 (7)	−0.0011 (5)	0.0023 (5)	0.0020 (5)
C3	0.0251 (7)	0.0154 (6)	0.0267 (7)	0.0002 (5)	0.0023 (5)	−0.0025 (5)
C4	0.0212 (6)	0.0114 (6)	0.0209 (6)	−0.0020 (5)	0.0020 (5)	0.0019 (5)
C5	0.0191 (6)	0.0173 (6)	0.0183 (6)	0.0031 (5)	0.0018 (5)	−0.0001 (5)
C6	0.0175 (6)	0.0193 (6)	0.0175 (6)	0.0045 (5)	0.0005 (5)	−0.0013 (5)
C7	0.0194 (6)	0.0250 (7)	0.0187 (6)	−0.0002 (5)	0.0004 (5)	−0.0006 (5)
C8	0.0292 (7)	0.0310 (8)	0.0233 (7)	0.0037 (6)	0.0055 (6)	0.0055 (6)
C9	0.0317 (8)	0.0398 (9)	0.0171 (6)	0.0156 (7)	−0.0001 (6)	0.0008 (6)
C10	0.0285 (7)	0.0394 (9)	0.0226 (7)	0.0094 (6)	−0.0078 (6)	−0.0121 (6)
C11	0.0226 (7)	0.0236 (7)	0.0257 (7)	0.0008 (5)	−0.0008 (5)	−0.0075 (6)
C12	0.0212 (6)	0.0202 (6)	0.0166 (6)	0.0002 (5)	−0.0008 (5)	0.0027 (5)

### Geometric parameters (Å, °)

**Table:**

O1—C3	1.4304 (17)	C5—H5A	0.9900
O1—C2	1.4310 (17)	C5—H5B	0.9900
N1—C12	1.5075 (16)	C6—C7	1.3943 (19)
N1—C4	1.5104 (18)	C6—C11	1.3946 (19)
N1—C1	1.5109 (16)	C7—C8	1.3914 (19)
N1—C5	1.5321 (16)	C7—H7	0.9500
C1—C2	1.5164 (19)	C8—C9	1.385 (2)
C1—H1A	0.9900	C8—H8	0.9500
C1—H1B	0.9900	C9—C10	1.381 (2)
C2—H2A	0.9900	C9—H9	0.9500
C2—H2B	0.9900	C10—C11	1.392 (2)
C3—C4	1.5138 (18)	C10—H10	0.9500
C3—H3A	0.9900	C11—H11	0.9500
C3—H3B	0.9900	C12—H12A	0.9800
C4—H4A	0.9900	C12—H12B	0.9800
C4—H4B	0.9900	C12—H12C	0.9800
C5—C6	1.5056 (17)		
			
C3—O1—C2	109.28 (10)	C6—C5—N1	116.35 (10)
C12—N1—C4	110.28 (10)	C6—C5—H5A	108.2
C12—N1—C1	110.87 (10)	N1—C5—H5A	108.2
C4—N1—C1	108.54 (10)	C6—C5—H5B	108.2
C12—N1—C5	105.42 (9)	N1—C5—H5B	108.2
C4—N1—C5	111.47 (9)	H5A—C5—H5B	107.4
C1—N1—C5	110.26 (10)	C7—C6—C11	118.87 (12)
N1—C1—C2	111.78 (11)	C7—C6—C5	119.38 (12)
N1—C1—H1A	109.3	C11—C6—C5	121.57 (12)
C2—C1—H1A	109.3	C8—C7—C6	120.61 (13)
N1—C1—H1B	109.3	C8—C7—H7	119.7
C2—C1—H1B	109.3	C6—C7—H7	119.7
H1A—C1—H1B	107.9	C9—C8—C7	120.04 (14)
O1—C2—C1	111.04 (11)	C9—C8—H8	120.0
O1—C2—H2A	109.4	C7—C8—H8	120.0
C1—C2—H2A	109.4	C10—C9—C8	119.76 (14)
O1—C2—H2B	109.4	C10—C9—H9	120.1
C1—C2—H2B	109.4	C8—C9—H9	120.1
H2A—C2—H2B	108.0	C9—C10—C11	120.56 (14)
O1—C3—C4	110.85 (11)	C9—C10—H10	119.7
O1—C3—H3A	109.5	C11—C10—H10	119.7
C4—C3—H3A	109.5	C10—C11—C6	120.15 (14)
O1—C3—H3B	109.5	C10—C11—H11	119.9
C4—C3—H3B	109.5	C6—C11—H11	119.9
H3A—C3—H3B	108.1	N1—C12—H12A	109.5
N1—C4—C3	111.52 (10)	N1—C12—H12B	109.5
N1—C4—H4A	109.3	H12A—C12—H12B	109.5
C3—C4—H4A	109.3	N1—C12—H12C	109.5
N1—C4—H4B	109.3	H12A—C12—H12C	109.5
C3—C4—H4B	109.3	H12B—C12—H12C	109.5
H4A—C4—H4B	108.0		
			
C12—N1—C1—C2	−70.18 (14)	C1—N1—C5—C6	−70.30 (14)
C4—N1—C1—C2	51.09 (13)	N1—C5—C6—C7	−93.35 (14)
C5—N1—C1—C2	173.45 (10)	N1—C5—C6—C11	91.63 (15)
C3—O1—C2—C1	61.86 (14)	C11—C6—C7—C8	−1.1 (2)
N1—C1—C2—O1	−57.37 (14)	C5—C6—C7—C8	−176.30 (12)
C2—O1—C3—C4	−62.50 (14)	C6—C7—C8—C9	0.2 (2)
C12—N1—C4—C3	70.00 (13)	C7—C8—C9—C10	0.6 (2)
C1—N1—C4—C3	−51.64 (13)	C8—C9—C10—C11	−0.5 (2)
C5—N1—C4—C3	−173.26 (11)	C9—C10—C11—C6	−0.5 (2)
O1—C3—C4—N1	58.57 (14)	C7—C6—C11—C10	1.3 (2)
C12—N1—C5—C6	169.98 (11)	C5—C6—C11—C10	176.32 (12)
C4—N1—C5—C6	50.32 (14)		

### Hydrogen-bond geometry (Å, °)

**Table:**

*D*—H···*A*	*D*—H	H···*A*	*D*···*A*	*D*—H···*A*
C3—H3A···Cl1^i^	0.99	2.70	3.6610 (14)	163
C5—H5A···Cl1^ii^	0.99	2.74	3.6304 (14)	150
C5—H5B···Cl1	0.99	2.63	3.5373 (14)	152
C9—H9···Cl1^iii^	0.95	2.80	3.5599 (16)	138
C12—H12A···Cl1^ii^	0.98	2.70	3.6085 (14)	155
C12—H12B···Cl1	0.98	2.78	3.6566 (14)	149
C12—H12C···Cl1^iv^	0.98	2.68	3.6380 (14)	166

Symmetry codes: (i) −*x*+1/2, *y*−1/2, *z*; (ii) −*x*+1/2, *y*+1/2, *z*; (iii) *x*+1/2, *y*, −*z*+3/2; (iv) *x*+1/2, −*y*+1/2, −*z*+1.
